# Cynomolgus monkey’s choroid reference database derived from hybrid deep learning optical coherence tomography segmentation

**DOI:** 10.1038/s41598-022-17699-7

**Published:** 2022-08-02

**Authors:** Peter M. Maloca, Christian Freichel, Christof Hänsli, Philippe Valmaggia, Philipp L. Müller, Sandrine Zweifel, Christine Seeger, Nadja Inglin, Hendrik P. N. Scholl, Nora Denk

**Affiliations:** 1 Institute of Molecular and Clinical Ophthalmology Basel (IOB), 4031 Basel, Switzerland; 2 Department of Ophthalmology, University Hospital Basel, 4031 Basel, Switzerland; 3 Moorfields Eye Hospital NHS Foundation Trust, London, EC1V 2PD UK; 4 Berner Augenklinik Am Lindenhofspital and University of Bern, Bern, Switzerland; 5 Department of Ophthalmology, University of Bonn, Bonn, Germany; 6 Makulazentrum Augsburg, Fachärzte Augenheilkunde, Augsburg, Germany; 7 University Hospital Zurich, Frauenklinikstrasse 24, 8091 Zurich, Switzerland; 8 University of Zurich, Rämistrasse 71, 8006 Zürich, Switzerland; 9 Pharma Research and Early Development (pRED), Pharmaceutical Sciences (PS), Roche, Innovation Center Basel, 4070 Basel, Switzerland

**Keywords:** Preclinical research, Imaging

## Abstract

Cynomolgus monkeys exhibit human-like features, such as a fovea, so they are often used in non-clinical research. Nevertheless, little is known about the natural variation of the choroidal thickness in relation to origin and sex. A combination of deep learning and a deterministic computer vision algorithm was applied for automatic segmentation of foveolar optical coherence tomography images in cynomolgus monkeys. The main evaluation parameters were choroidal thickness and surface area directed from the deepest point on OCT images within the fovea, marked as the nulla with regard to sex and origin. Reference choroid landmarks were set underneath the nulla and at 500 µm intervals laterally up to a distance of 2000 µm nasally and temporally, complemented by a sub-analysis of the central bouquet of cones. 203 animals contributed 374 eyes for a reference choroid database. The overall average central choroidal thickness was 193 µm with a coefficient of variation of 7.8%, and the overall mean surface area of the central bouquet temporally was 19,335 µm^2^ and nasally was 19,283 µm^2^. The choroidal thickness of the fovea appears relatively homogeneous between the sexes and the studied origins. However, considerable natural variation has been observed, which needs to be appreciated.

## Introduction

Cynomolgus monkeys are a commonly used species for preclinical research on ocular therapeutics, such as drug development or ocular gene therapy, given their close anatomical similarities to humans^1,2^.

In this context, optical coherence tomography (OCT) has been introduced as an adjunct investigation method to histopathological evidence to identify drug-related ocular toxicity in monkeys^3,4^. It is of great advantage that the retinas of cynomolgus macaques show structural analogies to those of humans—specifically the presence of a fovea, which can be depicted using OCT. The fovea centralis represents a depression located at the center of the macula, and since it is the site of the greatest density of photoreceptors (cones), it is responsible for the sharpest vision^5,6^. In fulfilling this purpose, the fovea is particularly susceptible, as it represents a region of comparatively increased metabolism^7–9^.

The fovea is a predestined site for hypoxic and neurodegenerative diseases. One possible reason may be due to its vascular deficits, as it is virtually completely dependent on adequate blood flow through the choroid^10,11^. It has been shown that the choroid supplies the outer retina with oxygen and nutrients and plays an essential role in its structural stability, waste removal, and heat dissipation^12,13^.

Given the fact that OCT imaging is used rather extensively in animal models and that, in an increasing number of cases, morphological OCT assessment is highly comparable to histopathology, the use of OCT as a constantly evolving imaging technique has been included in the framework of drug safety profiling^14,15^. In this context, it is noteworthy that significant differences in retinal thickness were found between Mauritius and Asian Macaques, despite being the same species but with different origins^16^. Although several studies have evaluated retinal and choroidal blood supply in macaques, few measurements have been conducted on a large number of individuals while taking into account both their origin and sex, thus providing appropriate reference data for research^17,18^. In addition, representative data of the natural variation of choroidal thickness are completely unknown.

Therefore, the primary goal of this study was to fill this important research gap and to provide a large reference choroid database for which an automated hybrid OCT deep learning method was established. This will allow for better analysis and comparability of the acquired choroid data.

## Materials and methods

### Animals and husbandry

A retrospective analysis of OCT data from studies conducted as part of routine pharmaceutical product development support was performed^19,20^. The purpose of these studies was to obtain OCT data on the safety assessment so that the animals were observed sequentially. Therefore, only OCT imaging data of untreated cynomolgus monkeys (*Macaca fascicularis*) of both sexes were collected in the current study. Thus, no additional animals were examined to obtain these data. The primary studies were reviewed and approved by the Institutional Animal Care and Use Committees (IACUC) of the respective institutions. Approval for the studies was granted by one of the following IACUCs: Charles River Laboratories Montreal, ULC Institutional Animal Care and Use Committee (CR-MTL IACUC), IACUC Charles River Laboratories Reno (OLAW Assurance No. D16-00594) and Institutional Animal Care and Use Committee (Covance Laboratories Inc., Madison, WI) (OLAW Assurance #D16-00137 (A3218-01). Within this study, animals were handled and used strictly according to the guidelines of the US National Research Council or the Canadian Council on Animal Care.

To ensure the animals’ safety and welfare, studies were reviewed and approved in advance by the Institutional Animal Care and Use Committees. The animals were bred specifically for laboratory use and obtained from certified suppliers in two geographical regions: Mauritius and Asia. The temperature of the room was kept constant between 20 °C and 26 °C; humidity was between 20 and 70%, with a 12:12 h light–dark cycle. Feeding was provided via a standard diet of pellets enriched with fresh fruits and vegetables. Clean and freely available tap water was provided and purified by reverse osmosis and UV irradiation. The animals were offered appealing psychological and environmental enrichment.

### OCT image data

Only OCT foveolar imaging data from healthy cynomolgus monkeys of Mauritian or Asian origin were included. These monkeys were between 30 and 50 months of age and had weights between 2.5 and 5.5 kg. OCT measurements were performed under anesthesia, as previously reported, with the pupil dilated using the Spectralis HRA + OCT Heidelberg device (Heidelberg Engineering, Heidelberg, Germany)^16^. The scanning protocol was the same for all animals and included a horizontal line scan pattern (centered over the fovea) with a size of 20° × 20°, consisting of 25 B-scans spaced 221 μm apart (scan length 5.3 mm, 512 × 496 pixels, scan depth 1.9 mm). The obtained images were exported from the OCT device as an original B-scan file in bitmap image data (BMP) format. Only image data with a scan quality of at least 25, provided by the manufacturer's software, was included.

### Image processing

**Figure 1:**
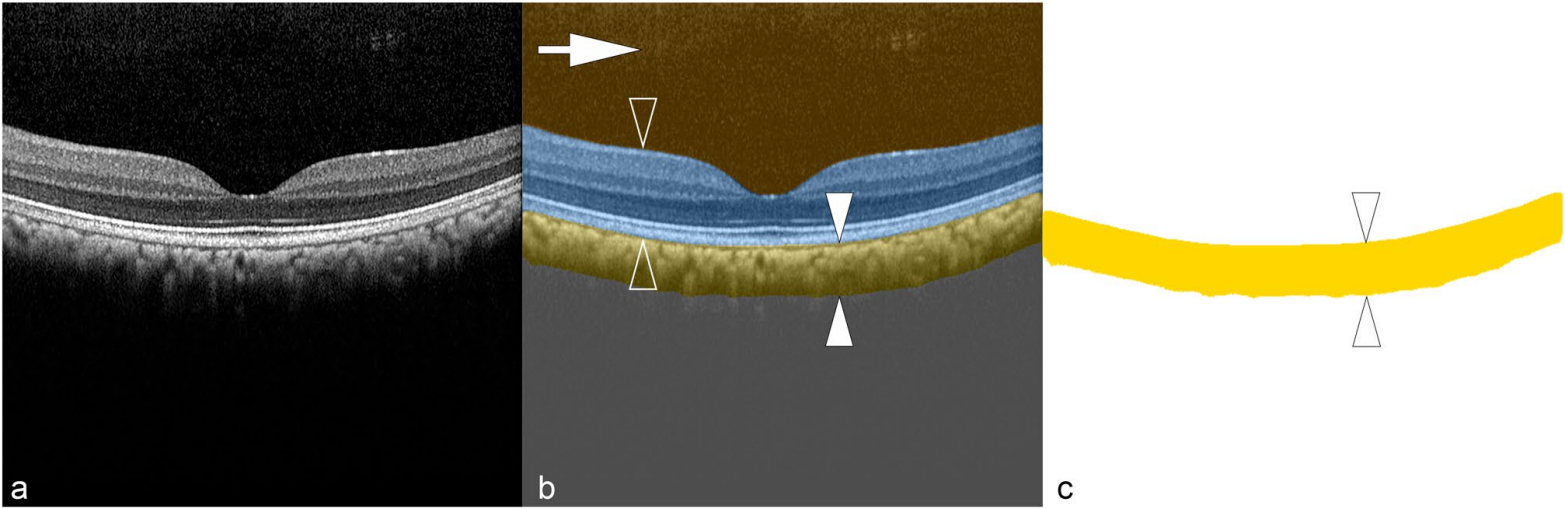
Illustration of automatic deep learning choroidal segmentation. (**a**) An original B-scan was exported from the device followed by (**b**) an automatic deep learning-based segmentation of the posterior eye into its main compartments. The deep learning predictions are displayed as overlays. The vitreous is highlighted in brown (arrow), the retina in blue (two open arrowheads), and the choroid in yellow (two white arrowheads), respectively. (**c**) Specific measurements were then made on the segmented choroid (yellow, two white arrowheads).

The obtained images were analyzed via two automatic processes (Fig. 1): (1) Using a previously developed and validated deep learning (DL) procedure, the OCT images were segmented into their corresponding compartments^16^, allowing the choroid to be segmented just above the choriocapillaris down to the choroid-sclera junction.

In summary the DL procedure used a modified U-Net architecture^21^, a type of convolutional neural network (CNN). Training and validation of the CNN was done using a representative subset of the OCT cynomolgus monkey data set^16^. This subset—the ground truth (GT)—contains 1100 B-scans obtained from 44 eyes from 44 individuals (each eye contributed 25 B-scans). GT annotation was done by three experienced retina specialists. Subsequently, the 44 eyes in the GT were randomly assigned to a training, validation, and test set containing 27, 9, and 8 eyes, respectively (675, 225, and 200 B-scans, respectively). Each human grader annotated 225 and 75 different B-scan for the training and validation sets. The 200 B-scans of the test set were annotated by each human grader (to investigate intergrader agreement of the ground truth labels). Data in the training set were augmented by applying vertical mirroring and adding a random rotation between − 8° and 8° degrees to each B-scan, increasing training set size to 2025 B-scans. On the test set, the differences between the CNN’s predictions and the annotations of the three human graders were, on average, smaller than the human intergrader differences. A detailed description of the ground truth annotation, CNN architecture, training, and evaluation is provided in Maloca et al.^22^.

(2) The second step of image processing was carried out by using a classical deterministic and structure-based computer vision algorithm to detect the deepest location within the fovea so that the whole approach can be described as hybrid image processing. This algorithm was implemented in C# (v7.0, .NET Framework v4.6). Because the internal limiting membrane (ILM) line extracted as the border between the segmentation of the vitreous and retinal compartments was rather noisy, the extracted ILM was smoothed using a moving average with a two-dimensional sampling window to determine the deepest point within the fovea. Thus, it was possible to automatically identify and define the deepest point of the fovea from the smoothed ILM, which was denoted as the nulla^16^. The nulla was therefore defined as the deepest position within a series of OCT B-scans of a particular macular OCT volume scan. This is particularly important because the nulla corresponds to the thinnest part of the fovea, where the receptors can interact most directly with light and which is commonly thought of as the place of sharpest vision. In the case of multiple deepest points (usually adjacent to each other), the coordinates of their center of mass were used as the deepest point.

**Figure 2:**
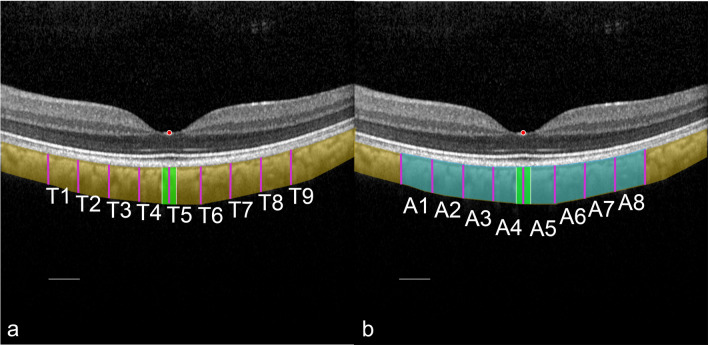
Designation of the anatomical choroidal landmarks in the left eye with relation to the deepest location of the foveola. (**a**) In a cross-sectional B-scan of a healthy macaque, the deepest location at the bottom was automatically identified and marked as a nulla (red dot). Below the nulla, consecutive measurements of choroidal thickness were conducted at 500 µm intervals up to 2000 µm to the side (marked as thickness T1–9, purple diameters). (**b**) In between the choroidal thickness diameters, the eight choroidal surface areas were defined (A1–A8, highlighted in light blue) and measured. With respect to the central bouquet of cones (highlighted in light green), umbo choroidal subfield analysis was similarly performed at distances of 100 µm to determine additional choroidal parameters for the umbo choroidal nasal and temporal thicknesses (a, white lines) and the umbo choroidal nasal and temporal surface areas (**b**). The same procedures were performed for all eyes. Bars = 500 µm.

Therefore, from the nulla as a reference point, an imaginary line was orthogonally projected to the underlying retinal pigment epithelium to measure the axial diameter of the choroid. Successive choroid measurements were carried out at distances of 500 µm to the side, up to a maximum distance of 2000 µm from the nulla^23,24^. This allowed the measurement of nine choroidal diameters (marked as thicknesses) in the axial direction, as well as eight of the intervening choroidal areas, yielding a total of 17 parameters for quantification of choroidal properties, as depicted in Fig. 2.

Given the importance of the nulla as the presumed site of the highest receptor density (central cone bouquet), further measurements of the choroid were made to determine whether a higher receptor density was also associated with a higher choroidal thickness^1,25^. Thus, the choroidal thickness and the intervening choroidal areas were measured laterally at an interval of 100 µm to the mentioned nulla. Thus, four more values were added: an additional nasal thickness (TUn) and a temporal thickness (TUt) in distance of 100 µm nasal and temporal to nulla, respectively, as well as an additional nasal choroid area (AUn) and a temporal choroid area (AUt). Including the choroidal thickness at the nulla itself, the nulla's sub-analysis provided a total of 5 parameters. Because of incomplete records, accurate data for the age and weight of monkeys were missing. This made it impossible to include these parameters in the analyses.

### Statistical analysis

For each of the measured thickness and area coefficients, the summary statistics—mean, standard deviation, minimum, and maximum—were calculated for subgroups of the data. Summary statistics were calculated for the left and right eyes separately, and boxplots were used to visualize the distribution of the data and the differences among subgroups (e.g., Mauritian versus Asian origin). Regarding the nulla, for the choroidal thickness (T5) and the areas of its adjacent choroidal surfaces (A4 and A5), the average mean values, minimum, maximum, and coefficient of variation (CV) were additionally calculated for all eyes. The CV was calculated as a relative measure of dispersion (defined as the ratio of the standard deviation to the mean). Pearson correlation coefficients were calculated among thickness and area coefficients. All calculations were performed in Python v3.8.5. Boxplots were generated using the Python library Seaborn v0.11.1. The impact of the categorical variables of sex (male, female) and origin (Mauritius, Asia) on each of the measured thickness coefficients was investigated by a two-way analysis of variance (ANOVA) using a type II sum-of-squares calculation. Adding the interaction term sex:origin to the ANOVA analyses did not change the significance levels of their results. Thus, the interaction terms were dropped. Since some monkeys contributed both left and right eyes, these eyes were not independent of each other and were analyzed separately. The 374 eyes contained 16 eyes of unknown origin, which were excluded from the ANOVA analyses. ANOVA was performed using the Python library statsmodels v0.12.1. The significances of the differences among group means were calculated using the F statistic, which is part of statsmodels’s ANOVA implementation. Bonferroni correction of significance levels was applied to adjust for the multiple testing problem by dividing significance levels by nine, the number of statistical tests per eye.

## Results

### General results

Retinal scans of 374 eyes from 203 animals and from eight different studies were analyzed retrospectively. Females contributed 147 eyes (39.30%) and males 227 (60.70%), with 186 being left eyes (49.73%) and 188 (50.27%) being right eyes. There were 199 eyes (53.21%) from animals from Mauritius and 159 (42.51%) from animals from Asia. Sixteen eyes were of unknown origin, which were not included in the ANOVAs of the nulla sub-analysis.

### Overall analysis

The overall average choroidal thickness at the nulla was 192.83 µm (ranging from 148.20 µm to 269.10 µm with a coefficient of variation (CV) of 7.8%). The overall mean central bouquet temporal surface area was 19,335 µm^2^ (ranging from 14,792 µm^2^ to 27,936 µm^2^ with a CV of 8.2%) and the nasal surface area was 19,283 µm^2^ (ranging from 15,386 µm^2^ to 27,343 µm^2^ with a CV of 8.3%).

### Correlation analysis

The results of the Pearson correlation analysis are summarized in Table 1. The correlation analysis revealed a relatively high correlation between adjacent thickness coefficients (0.67–0.77, Table 1a). Between non-adjacent thickness coefficients, the correlation is smaller (0.40–0.72, Table 1a). In terms of statistical hypothesis testing, it is thus plausible to analyze the nine thickness coefficients separately, even though there is some correlation among them and *p*-values might not be entirely reliable. On the other hand, the eight area coefficients (A1–A8) are highly correlated with the thickness coefficients (0.85–0.88, Table 1b). The coefficients of the nulla sub-analysis (TUn, TUt, AUn, AUt) are all highly correlated with T5 (0.90–0.91, Table 1b). In terms of statistical hypothesis tests, it is thus sufficient to analyze just T1–T9, excluding A1–A8 and the four coefficients of the nulla sub-analysis (TUn, TUt, AUn, AUt).

**Table 1:** Pearson correlation coefficients (A) among thickness coefficients and (B) between thickness and area/nulla sub-analysis coefficients.

	T1	T2	T3	T4	T5	T6	T7	T8	T9			
**(a)**												
T1		0.70	0.57	0.60	0.54	0.56	0.55	0.52	0.40			
T2			0.71	0.68	0.61	0.63	0.60	0.57	0.46			
T3				0.77	0.61	0.67	0.56	0.58	0.46			
T4					0.67	0.72	0.61	0.59	0.51			
T5						0.69	0.66	0.61	0.54			
T6							0.74	0.64	0.57			
T7								0.69	0.61			
T8									0.69			
T9												
**(b)**												
Var1	T1	T2	T3	T4	T6	T7	T8	T9	T5	T5	T5	T5
Var2	A1	A2	A3	A4	A5	A6	A7	A8	TUn	TUt	AUn	AUt
r	0.87	0.87	0.88	0.85	0.88	0.86	0.86	0.87	0.90	0.91	0.91	0.90

*Var1* variable 1, *Var2* variable 2, *r* Pearson correlation coefficient.

### Subgroup results

**Figure 3:**
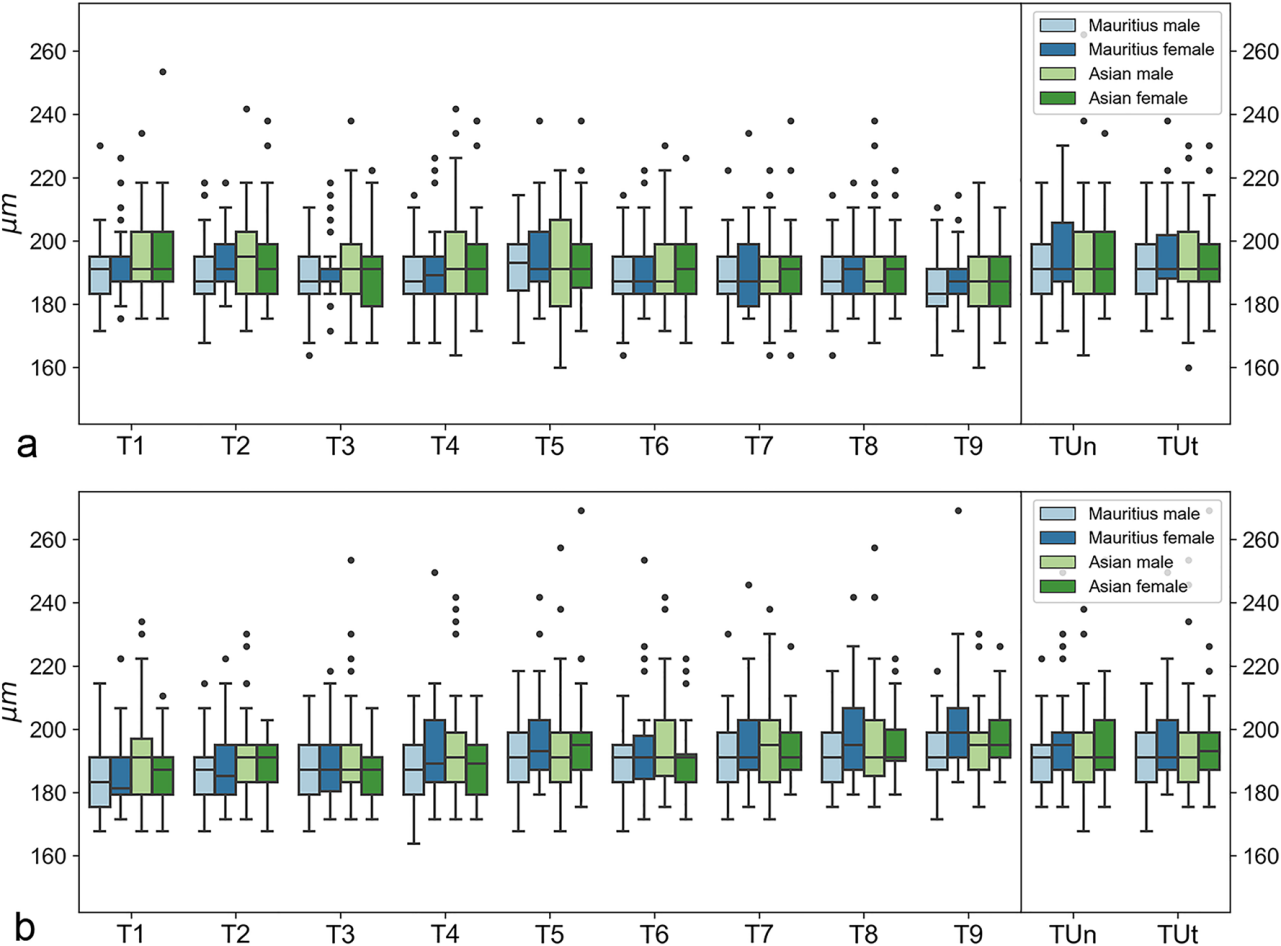
Boxplots of sex-specific and origin-specific variations in choroidal thickness for right (**a**) and left (**b**) eyes. Numerical data of Mauritius male, Mauritius female, Asian male, and Asian female are plotted for each thickness coefficient. Rectangular boxes represent interquartile ranges (IQR), which extend from Q1 to Q3. Black lines in the middle of IQR indicate medians. Upper whiskers extend to the last datum, which is smaller than Q3 + 1.5 × IQR. Lower whiskers extend to the first datum, which is greater than Q1 − 1.5 × IQR. Data beyond whiskers are outliers and plotted as black circles.

**Figure 4:**
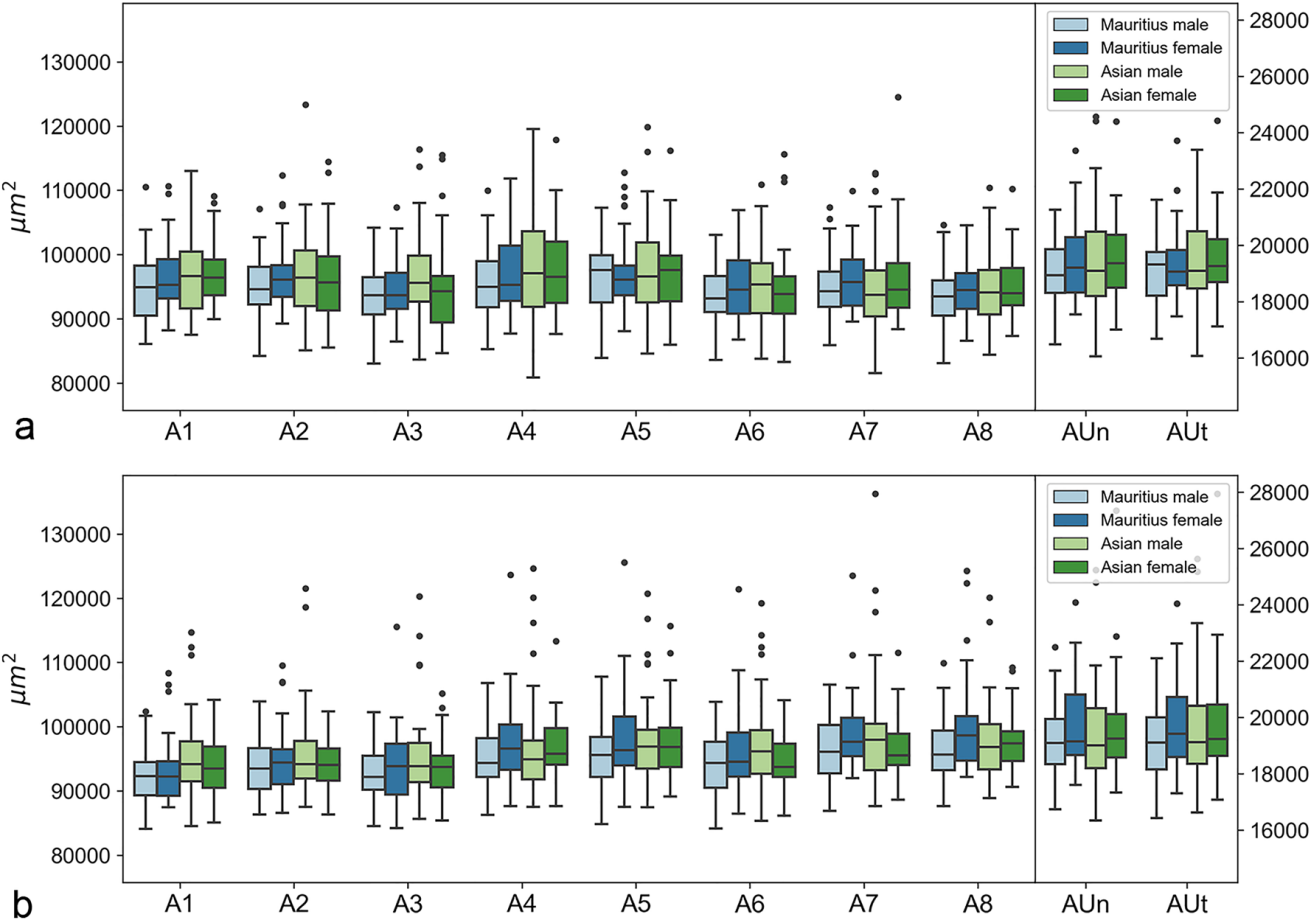
Boxplots of sex-specific and origin-specific variations in choroidal areas for right (**a**) and left (**b**) eyes. Numerical data of Mauritius male, Mauritius female, Asian male, and Asian female are plotted for each area coefficient.

The results in relation to sex, origin, and eye side are summarized in Figs. 3 and 4 and in Tables 2 and 3.

**Table 2:** Summary statistics of the choroid thickness values.

	Stats	Sex	Origin	T1	T2	T3	T4	T5	T6	T7	T8	T9	TUn	TUt
OD	Mean	Male	Mauritius	190	190	189	190	192	189	189	188	186	190	193
	Std	Male	Mauritius	10	11	10	11	11	10	10	9	10	11	11
	Min	Male	Mauritius	172	168	164	168	168	164	168	164	164	168	172
	Max	Male	Mauritius	230	218	211	215	215	215	222	215	211	218	218
OD	Mean	Male	Asian	195	194	193	194	192	192	190	191	188	194	194
	Std	Male	Asian	13	14	14	16	17	14	13	15	13	20	15
	Min	Male	Asian	176	172	168	164	160	172	164	168	160	164	160
	Max	Male	Asian	234	242	238	242	222	230	222	238	218	265	230
OD	Mean	Female	Mauritius	192	194	192	191	195	192	191	191	188	195	195
	Std	Female	Mauritius	11	10	10	13	14	11	12	10	9	13	14
	Min	Female	Mauritius	176	179	172	168	176	176	176	176	172	172	176
	Max	Female	Mauritius	226	218	218	226	238	222	234	218	215	230	238
OD	Mean	Female	Asian	196	194	190	192	194	190	190	190	188	194	194
	Std	Female	Asian	14	14	14	16	14	13	13	10	11	14	12
	Min	Female	Asian	176	176	168	172	172	168	164	176	168	176	172
	Max	Female	Asian	254	238	222	238	238	226	238	222	211	234	230
OS	Mean	Male	Mauritius	185	187	187	188	192	189	191	193	193	191	191
	Std	Male	Mauritius	9	10	9	11	11	10	11	11	9	10	12
	Min	Male	Mauritius	168	168	168	164	168	168	172	176	172	176	168
	Max	Male	Mauritius	215	215	211	211	218	211	230	218	218	222	215
OS	Mean	Male	Asian	192	192	192	193	194	194	196	197	195	192	196
	Std	Male	Asian	15	13	16	17	18	16	15	18	11	15	17
	Min	Male	Asian	168	172	172	172	168	176	172	176	176	168	176
	Max	Male	Asian	234	230	254	242	257	242	238	257	230	238	254
OS	Mean	Female	Mauritius	186	188	189	193	197	195	196	199	202	196	196
	Std	Female	Mauritius	12	11	11	15	14	16	14	14	16	16	14
	Min	Female	Mauritius	172	172	172	172	179	172	176	179	183	176	179
	Max	Female	Mauritius	222	222	218	250	242	254	246	242	269	250	250
OS	Mean	Female	Asian	186	188	187	189	196	191	194	195	198	195	196
	Std	Female	Asian	10	9	8	9	17	10	11	11	11	15	17
	Min	Female	Asian	168	168	172	172	176	172	179	179	183	176	176
	Max	Female	Asian	211	203	207	211	269	222	226	222	226	254	269

*OD* oculus dexter, *OS* oculus sinister, *Stats* statistical analysis, *T* thickness, *U* umbo, *n* nasal, *t* temporal, *std* standard deviation, *min* minimum, *max* maximum, values in µm.

**Table 3:** Summary statistics of the choroidal area values.

	Stats	Sex	Origin	A1	A2	A3	A4	A5	A6	A7	A8	AUn	AUt
OD	Mean	Male	Mauritius	94,653	94,794	93,639	95,476	96,334	93,540	94,769	93,059	19,063	19,160
	Std	Male	Mauritius	4907	4382	4840	5366	5169	4371	4551	4210	1208	1200
	Min	Male	Mauritius	86,104	84,216	83,052	85,261	83,893	83,594	85,893	83,113	16,473	16,683
	Max	Male	Mauritius	110,548	107,101	104,187	109,953	107,305	103,084	107,377	104,614	21,262	21,620
OD	Mean	Male	Asian	96,692	96,547	96,942	97,749	97,869	95,215	95,180	94,365	19,399	19,443
	Std	Male	Asian	6182	6803	6624	7950	7491	5627	6892	5691	1908	1681
	Min	Male	Asian	87,530	85,103	83,625	80,820	84,590	83,743	81,537	84,363	16,051	16,071
	Max	Male	Asian	113,010	123,321	116,359	119,554	119,881	110,913	112,694	110,429	24,564	23,383
OD	Mean	Female	Mauritius	96,517	97,101	94,474	97,287	97,552	94,969	96,026	94,930	19,409	19,364
	Std	Female	Mauritius	5471	5364	5188	5858	6167	5337	4758	4278	1407	1354
	Min	Female	Mauritius	88,220	89,235	86,419	87,716	88,049	86,734	89,550	86,586	17,543	17,471
	Max	Female	Mauritius	110,653	112,355	107,374	111,834	112,741	106,938	109,923	104,556	23,359	23,707
OD	Mean	Female	Asian	97,151	96,404	94,877	97,115	96,751	94,466	96,109	95,301	19,460	19,464
	Std	Female	Asian	4834	6553	7250	6657	6057	6694	6678	4832	1453	1401
	Min	Female	Asian	89,943	85,488	84,638	87,605	85,919	83,251	88,403	87,314	17,005	17,110
	Max	Female	Asian	109,118	114,424	115,483	117,893	116,207	115,652	124,525	110,205	24,397	24,416
OS	Mean	Male	Mauritius	92,130	93,627	93,074	95,328	95,784	94,142	96,427	96,297	19,144	19,149
	Std	Male	Mauritius	3982	4254	4267	4739	4846	4832	5014	4514	1187	1222
	Min	Male	Mauritius	84,096	86,347	84,493	86,243	84,802	84,128	86,880	87,620	16,736	16,417
	Max	Male	Mauritius	102,356	103,905	102,273	106,812	107,756	103,886	106,525	109,902	22,495	22,094
OS	Mean	Male	Asian	95,397	95,816	95,542	97,233	97,972	97,338	99,004	97,698	19,436	19,615
	Std	Male	Asian	6547	7161	7017	8379	7272	7372	9406	6486	1834	1950
	Min	Male	Asian	84,545	87,510	85,663	87,477	87,419	85,299	87,632	88,856	16,343	16,616
	Max	Male	Asian	114,698	121,559	120,317	124,616	120,756	119,208	136,261	120,087	25,225	25,635
OS	Mean	Female	Mauritius	93,252	94,549	94,048	97,885	98,167	96,351	99,173	100,234	19,770	19,755
	Std	Female	Mauritius	5197	5342	5720	6903	6788	6449	5937	7329	1545	1499
	Min	Female	Mauritius	87,422	86,541	84,208	87,642	87,516	86,429	92,011	92,181	17,597	17,297
	Max	Female	Mauritius	108,354	109,520	115,539	123,680	125,572	121,387	123,546	124,291	24,079	24,040
OS	Mean	Female	Asian	93,559	94,014	93,726	96,520	97,710	94,768	96,819	98,100	19,648	19,764
	Std	Female	Asian	4349	3742	4311	5070	5775	4192	4630	4669	1774	1933
	Min	Female	Asian	85,078	86,318	85,363	87,628	89,102	86,139	88,621	90,620	17,335	17,073
	Max	Female	Asian	104,207	102,373	105,145	113,355	115,707	104,130	111,532	109,202	27,343	27,936

*OD* oculus dexter, *OS* oculus sinister, *Stats* statistical analysis, *A* choroid surface area, *U* umbo, *n* nasal, *t* temporal, *std* standard deviation, *min* minimum, *max* maximum, values in µm^2^.

The observed variability does not appear to depend on sex, origin, or their interaction; this was confirmed by statistical hypothesis tests based on ANOVA analyses. For each of the thickness coefficients T1–T9, a statistical hypothesis test was performed to test whether the independent variables sex and/or origin affected the observed variability in that thickness coefficient. No significant effects were detected in the right eyes. In the left eyes, only for T9 was a significant effect detected for sex, with a *p*-value of 0.00126. To adjust for the multiple testing problem, Bonferroni correction was applied by dividing the significance levels by nine (the number of statistical tests per eye). This caused the *p*-value of 0.00126 to fall into the uncorrected significance level 0.01 < α < 0.05, because 0.01/9 = 0.00111 < 0.00126 < 0.05/9 = 0.00556. Thus, this effect is weakly significant, potentially indicating a false positive.

In summary, the choroid was relatively uniform in terms of foveolar depression across all monkeys.

## Discussion

Due to genetic and anatomical similarities to humans, cynomolgus monkeys have emerged as an ideal model for a number of innate and acquired retinal diseases^3,26–28^. Cynomolgus monkeys have also been found to exhibit soft drusen comparable to human early age-related macular degeneration, thereby offering insights into drusen biogenesis^29^. In another cynomolgus monkey family, retinal degeneration with cystoid macular edema was observed, which is typical for retinitis pigmentosa (RP), so this model might be useful for studies on the mechanism of disease pathogenesis or the evaluation of new treatments with respect to specific retinal degeneration^30^.

The fovea is characterized by the highest concentration of cones, which enables the sharpest vision^5^. In contrast to the extraordinary high metabolic performance, the foveolar cones are located at the greatest distance from the retinal vessels, such that this extraordinary avascularity turns the fovea primarily hypoxic^31,32^. This potential imbalance between demand and supply can only be compensated by sufficient supply from the choroid, such that the central fovea is one hundred percent dependent on the choroid^13^.

Despite the paramount role of the choroid in the fovea, there is a substantial deficiency in the current literature regarding reference choroidal values in cynomolgus monkeys. Therefore, this study focused on the normal range of cynomolgus monkey eyes in order to fill this knowledge gap for the first time using automated image processes on an unprecedented number of eyes.

Interestingly, it was found that the most central parts of the choroid (and thus the closest to the foveolar cones) were relatively homogeneously structured across all cynomolgus monkeys and did not seem to be affected by origin or sex. Besides, a relatively low level of dispersion was revealed with coefficients of variation between 7.8% and 8.3%. Ideally, a correlation with the age of the animals or eye axis length could be considered to better understand this interesting variation; unfortunately, such data were not available in this retrospective study, so this will have to be investigated in the future. The measured choroidal values were in complete contrast with observations of the architecture of the retina of the same study population^16^. Thus, the central choroid showed a certain conservation of its structural blueprint and appears to be independent of sex and origin. There is presumably a global and unified choroidal design that is maintained across sexes and origins to provide the fovea with nutrients and adequate metabolites. The obtained values suggest that readings for the central choroid can be used interchangeably, in contrast to the paracentral domains. For reference, alle results of the current study are shown in Supplementary Table S1.

Overall, the patterns of variability seem very similar across all measurements, T1–T9 and A1–A8. In relation to the central and quite homogeneous choroid, an inverse relation was found over all eyes when considering the paracentral choroid. Here, a minor variation was detected over all eyes. Despite all similarities, the values show that the subfoveal choroid is significantly thinner in the cynomolgus monkey compared to humans, even up to 150 µm^33–35^. The segmentation of the choroid by deep learning depends on the ground truth quality generated by human graders. Therefore, the current segmentations should be considered with caution. However, the deviation among human graders in a previous study with comparable data was lower than compared to the DL algorithm^16^.

A possible limitation is that a relatively rigid pattern was used for choroidal data analysis. For example, the angle of the measurement lines was set to a strict rectangular grid without considering individual deviations with respect to the retinal pigment epithelium^34^. Another limitation was that the exact age was not assessed so that an age correlation was not possible. Nevertheless, the values for this age group are representative^36^. No consideration was given to diurnal variations, which potentially could be as high as 30 µm^37,38^. Unfortunately, the refractive status was not measured as this was not the aim of the previous investigations. Axial length measurements were not performed. Thus, correction for the ocular magnification factor was not feasible^39,40^. However, this topic is under discussion, and an internationally recognized consensus does not yet exist at the time of writing^41^. Another limitation was that the outer delineation of the transition between the choroid and the sclera was challenging to define in the initial deep learning training due to the relatively intense choroidal pigmentation, as illustrated in Fig. 2 of Maloca et al.^22^. Therefore, it is possible that the identified location of the effective boundary was not pixel-precisely identical to its physical location, which could lead to slight error. However, it would not have been possible to surgically separate the choroid and superimpose these manually segmented boundaries. Nevertheless, the artificial neural network training showed quite good agreement to human annotations^40^. In future studies, however, this circumstance needs to be further investigated. Another limitation may be that in the previously used scan protocol, the distances between the B-scan were relatively too large, so that a certain uncertainty regarding the exact localization of nulla could be induced. However, the scan resolution will inevitably be improved in future studies. The results were obtained from only one OCT device, so that a comparison with other OCT systems is missing. Since differences between the OCT devices are known, the results should be considered with caution. However, a comparison between different devices was not the aim of this study.

## Conclusions

In summary, using an advanced hybrid deep learning approach, we succeeded in generating objective values for a reference choroid database derived from an unprecedented number of cynomolgus monkeys’ eyes. This revealed a relatively uniform blueprint for the central choroidal architecture, regardless of origin or sex, which is interlinked to the foveal photoreceptors (cones). Notable is also the large sample size used in this study, which generally leads to more reliable results with greater precision and statistical power compared to studies done with a smaller number of eyes. Thanks to the large number of eyes, it was nevertheless possible to discover a noteworthy natural variation. This suggests a cautious interpretation of choroidal thickness measurements. Thus, when assessing findings, it is important to bear in mind that a supposed pathology could merely represent individuality. Therefore, the provided data are essential for describing the natural course of choroidal conditions and evaluating the adverse effects of drugs in preclinical safety studies.

## Supplementary Information

Supplementary Table S1.

## Data Availability

All relevant data are presented within this paper and its supporting information. All further information can be obtained on request from the corresponding author.
